# Systematic review of risk prediction studies in bone and joint infection: are modifiable prognostic factors useful in predicting recurrence?

**DOI:** 10.5194/jbji-6-257-2021

**Published:** 2021-07-08

**Authors:** Maria Dudareva, Andrew Hotchen, Martin A. McNally, Jamie Hartmann-Boyce, Matthew Scarborough, Gary Collins

**Affiliations:** 1 Centre for Statistics in Medicine, Nuffield Department of Orthopaedics, Rheumatology and Musculoskeletal Sciences, University of Oxford, Oxford, UK; 2 Division of Trauma and Orthopaedic Surgery, Addenbrooke's Hospital, Cambridge University Hospitals, Cambridge, UK; 3 Bone Infection Unit, Nuffield Orthopaedic Centre, Oxford University Hospitals NHS Foundation Trust, Oxford, UK; 4 Centre for Evidence-Based Medicine, Nuffield Department of Primary Care Health Sciences, University of Oxford, Oxford, UK

## Abstract

**Background**: Classification systems for orthopaedic infection include patient health status, but there is no consensus about which comorbidities affect prognosis. Modifiable factors including substance use, glycaemic control, malnutrition and obesity may predict post-operative recovery from infection. **Aim**: This systematic review aimed (1) to critically appraise clinical prediction models for individual prognosis following surgical treatment for orthopaedic infection where an implant is not retained; (2) to understand the usefulness of modifiable prognostic factors for predicting treatment success. **Methods**: EMBASE and MEDLINE databases were searched for clinical prediction and prognostic studies in adults with orthopaedic infections. Infection recurrence or re-infection after at least 6 months was the primary outcome. The estimated odds ratios for the primary outcome in participants with modifiable prognostic factors were extracted and the direction of the effect reported. **Results**: Thirty-five retrospective prognostic cohort studies of 92 693 patients were included, of which two reported clinical prediction models. No studies were at low risk of bias, and no externally validated prediction models were identified. Most focused on prosthetic joint infection. A positive association was reported between body mass index and infection recurrence in 19 of 22 studies, similarly in 8 of 14 studies reporting smoking history and 3 of 4 studies reporting alcohol intake. Glycaemic control and malnutrition were rarely considered. **Conclusion**: Modifiable aspects of patient health appear to predict outcomes after surgery for orthopaedic infection. There is a need to understand which factors may have a causal effect. Development and validation of clinical prediction models that include participant health status will facilitate treatment decisions for orthopaedic infections.

## 1 Introduction

Risk assessment plays a pivotal role in clinical decision-making. Clinical prediction models combine prognostic factors into an equation to estimate the probability of a patient experiencing a health outcome in the future. Models can also predict response to a treatment, exacerbation of a condition, or an adverse event such as mortality (Grant, 2018). Clinical prediction models may be used to decide the setting of care (NICE QS110, 2016), whether or not a diagnostic test should be used (Stiell, 1992) or a particular treatment should be offered (NICE CG181, 2016), and to communicate risk around clinical decisions (Wishart et al., 2010).

Clinical prediction models can be used to estimate the success of bone and joint infection treatment according to a patient's baseline health. This has been applied following debridement, antibiotics and implant retention (DAIR) for prosthetic joint infection (PJI) (Duffy et al., 2018; Wouthuyzen-Bakker et al., 2019).

Existing classification systems for orthopaedic infections differ in what health factors they include. The McPherson classification of PJI uses the number of comorbidities to stratify host health; however, why particular comorbidities were chosen is unclear (McPherson, 2002). The validation of host classification in the McPherson system detected no association between host status and treatment outcome in 50 patients. Host status was associated with the “number of surgical complications”, which included urinary retention, thrombocytopaenia and non-allergic antimicrobial reactions ranked equally with stroke, septic shock, respiratory failure and death.

The BACH stratification tool for long bone osteomyelitis has recently been developed and validated, categorising distribution of infection within a bone, antibiotic options, the management of the soft tissue required for wound closure, and host status. Host status is classified as favourable (H1) or unfavourable (H2), with some suggested comorbidities conferring H2 class. Discrepancies were observed in how H2 was interpreted by clinicians (Hotchen et al., 2019). The Cierny–Mader classification of long bone osteomyelitis divides adverse host factors into local and systemic compromising conditions (Mader et al., 1997). It specifies “tobacco abuse, i.e. > 2 packs d${}^{-1}$” as the cut-off for local compromise but does not describe how this was derived. Phrases such as “major vessel compromise” were not defined and may be open to interpretation.

Modifiable prognostic factors affecting health, such as malnutrition, blood glucose control, smoking and alcohol use, can affect healing and immunity. Interventions addressing these factors can improve outcomes after surgery (Norman et al., 2008; Thomsen et al., 2014; Barr et al., 2016; Hopkins et al., 2017). Prognostic modelling studies could help identify whether these modifiable prognostic factors predict successful treatment for bone and joint infections. If accurate, validated clinical prediction models that include comorbidities and modifiable prognostic factors already exist, they could inform the “host status” section of classification systems such as BACH. This would help to (a) select treatment for bone and joint infections; (b) discuss with patients the likelihood of a successful treatment outcome as part of valid informed consent; (c) provide a benchmark for expected rates of treatment success; and (d) select participants for investigation of treatment methods.

Important outcomes for patients considering orthopaedic surgery include pain, mobility and independence, fear (including of sepsis and severe illness), sleep quality, work, social function, and the burden of treatment (Trickett et al., 2012; Baumhauer et al., 2013; Moore et al., 2015). Generic patient-reported outcome measures (PROMS) contain some of these measures and may be particularly useful when consensus criteria for infection eradication are not available (Lipsky et al., 2004; Baumhauer et al., 2013; Diaz-Ledezma et al., 2013; Metsemakers et al., 2018).

The development, reporting and validation of clinical prediction models have been extensively reviewed. Guidance is available for publication and assessing reporting quality (Hayden et al., 2013; Moons et al., 2014, 2015; Collins et al., 2015; Grant et al., 2018; Wolff et al., 2019). TRIPOD guides the reporting of studies describing the creation and validation of clinical prediction models (Collins et al., 2015; Moons et al., 2015). Different methodological assumptions apply to prediction modelling and analysis for causal inference.

The primary objective of this study was to systematically review and critically appraise clinical prediction models that included patient comorbidities (particularly modifiable prognostic factors), developed for the prognosis of surgically treated musculoskeletal infection. We aimed to find models that included substance use (at least smoking and alcohol intake), hyperglycaemia, malnutrition, and obesity.

An additional objective was to systematically review prognostic studies that did not fit the definition of clinical prediction modelling to identify the direction of association between potentially modifiable prognostic factors and treatment success. The aim of this analysis was to identify the usefulness of modifiable factors for prognostic modelling, rather than causal inference.

## 2 Methods

A systematic review (PROSPERO CRD42020177814) was conducted to evaluate published studies reporting epidemiological or prognostic modelling of orthopaedic infection recurrence after curative surgery (including removal of infected implants and debridement) in adults.

EMBASE and MEDLINE databases were searched from inception to July 2020. Reference lists from studies identified during the review were searched. Search terms for diagnoses included synonyms for spondylodiscitis, osteomyelitis, fracture-related infection, and prosthetic joint infection. Terms for surgical treatment and outcome were included. Synonyms for variables relating to smoking, alcohol use, hyperglycaemia, malnutrition, and obesity were included, chosen based on preliminary searches in November 2018. Search strings are presented in the Supplement.

The following studies were excluded: case-control studies unadjusted for population outcome risk; patients treated without surgery or without implant removal; children < 18 years old; and those that did not include follow-up of at least 6 months.

The main outcome was recurrence of orthopaedic infection or new orthopaedic infection at the same anatomic site, defined using any criteria, including composite outcomes. This is referred to as “treatment failure”. PROMS were considered a secondary treatment outcome.

Studies were identified as risk prediction modelling studies if they reported a multivariable (two or more predictors) risk prediction model for treatment failure. Additionally, the aims, statistical modelling methods, model interpretation, intended use, and validation reported were assessed.

Data extracted included the study design, participant selection and loss, sample size, predictor selection and measurement; outcome incidence, definition and ascertainment; and modelling considerations. The latter included missing data handling, the choice of statistical model, whether assumptions were violated, and the handling of competing outcomes such as participant death or limb amputation. For clinical prediction modelling, model shrinkage for overoptimism, model calibration and discrimination and validation were recorded.

For clinical prediction studies, applicability and risk of bias were assessed using the PROBAST tool. For epidemiologic studies, domains from the QUIPS tool and CHARMS checklist were used to ascertain study relevance and stratify risk of bias from low to high across methodological domains (Hayden et al., 2013; Moons et al., 2014, 2015; Collins et al., 2015; Wolff et al., 2019). Appropriateness of sample size was assessed according to the number of listed predictors specified in the methods, or, if this was not available, predictors listed in univariable analysis. The minimum appropriate sample size was estimated using the pmsampsize package for R, assuming an estimated $R^{2}$ of 0.25 (selected to be generous) and parameters from the study report (Ensor et al., 2019). Assuming an estimated average orthopaedic infection recurrence rate of 20 % at least 12 months following treatment, the maximum attainable Cox–Snell $R^{2}$ is 0.63.

Data were extracted from studies by two researchers in parallel (Maria Dudareva, Andrew Hotchen). Risk of bias was assessed across domains independently. Disagreements were resolved by consensus with supervision from a third researcher if required (Gary Collins).

Due to the anticipated heterogeneity of studies following a preliminary search in November 2018, guidance for synthesis without meta-analysis (SWIM) was followed for summary measures for prognostic factors of interest (Campbell et al., 2020).

The direction of association for each prognostic variable was determined. This was defined as positive (point estimate of odds ratio of treatment failure > 1 for participants with the prognostic factor), negative (point estimate of odds ratio of treatment failure < 1 for participants with the prognostic factor), or null (estimated odds ratio equal to 1). Adjusted values were preferentially recorded, if this included the prognostic factor of interest. Unadjusted odds ratios, if not reported, were estimated from baseline characteristics or published datasets.

Analyses were performed using R version 3.6.3 (R Core Team, 2020).

**Figure 1:**
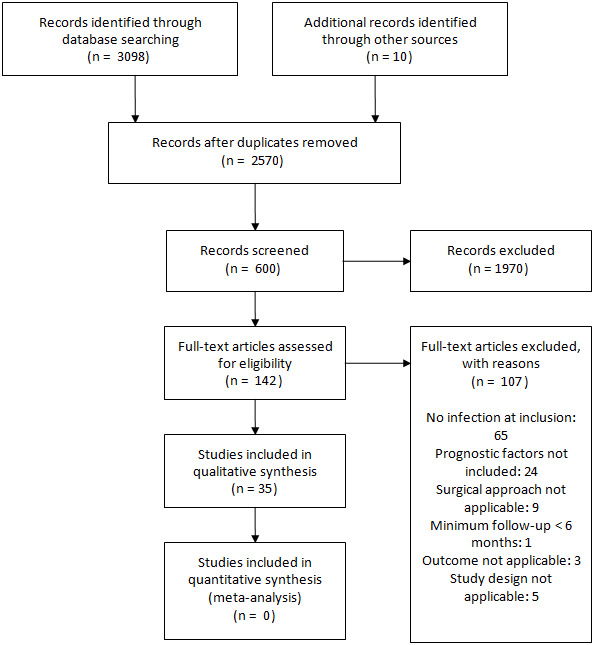
PRISMA 2009 study inclusion flowchart.

**Table 1:** Characteristics of participants and design of included studies. Italicised studies did not describe multivariable prognostic modelling and were considered epidemiologic studies for the purposes of analysis.

Study	Design	Diagnoses	Treatment
*Ahmad et al. (2019)*	*Retrospective cohort studySurvival analysis*	*PJI of primary total hip arthroplasty*	*Two-stage revision arthroplasty*
*Anderson etal.(2018)*	*Retrospective multi-centre cohortstudyUnivariable analysis*	*PJI of total knee arthroplasty. MSIS definition of PJI (Parvizi, 2014)*	*Revision arthroplasty with extensor mechanism reconstruction*
Barshes et al.(2016)	Retrospective cohort study Cox regression survival analysis	Foot osteomyelitis without orthopaedic implants; clinical diagnosis with 91 % positive histology	Surgical debridement of osteomyelitis in all but 15 participants
Barton et al.(2019)	Retrospective cohort study Logistic regression	PJI of total hip arthroplasty. MSIS definition of PJI (Parvizi, 2014)	Two-stage revision arthroplasty
Bejon et al.(2010)	Retrospective cohort study Cox regression survival analysis	PJI defined by clinical diagnosis	Two-stage revision arthroplasty
Cancienne et al. (2017)	PearlDiver Medicare database multicentre retrospective cohort Logistic regression	PJI of total hip arthroplasty defined by ICD-9 code and CPT procedure codes	Two-stage revision arthroplasty
Cancienne et al. (2018)	PearlDiver Medicare database multicentre retrospective cohort Logistic regression	Participants > 65 years old PJI of total knee arthroplasty defined by ICD-9 code and CPT procedure codes	Two-stage revision arthroplasty
*Carrega et al. (2020)*	*Retrospective cohort studyUnivariable analysis*	*MSIS definition of PJI (Parvizi, 2014)*	*Two-stage revision arthroplasty*
Cha et al.(2015)	Retrospective cohort study Logistic regression	PJI of total knee arthroplasty. MSIS definition of PJI (Parvizi, 2014)	Two-stage revision arthroplasty
Chen et al.(2017)	Retrospective cohort study Cox regression survival analysis	PJI of total knee arthroplasty. Defined by any of: sinus, purulence, >= 2 positive cultures, histopathology, abnormal CRP and ESR.	Two-stage revision arthroplasty
Cochran et al.(2016)	100 % Medicare Part A multicentre retrospective cohort study Cox regression survival analysis	PJI of primary total knee arthroplasty defined by ICD-9-CM code 996.66	Incision and drainage with or without liner exchange, single-stage and two-stage revision arthroplasty
*Cook et al.(2007)*	*Retrospective cohort study*	*Calcaneal osteomyelitis defined by surgical treatment*	*Partial calcanectomy*
*Faschingbauer et al. (2019)*	*Retrospective cohort study*	*PJI of primary total knee arthroplasty (not defined)*	*First two-stage revision arthroplasty*
*Ford et al.(2018)*	*Retrospective cohort study*	*PJI of total hip or knee arthroplasty defined by ICD-9-CM code 996.66*	*First two-stage revision arthroplasty*
Garcia delPozo et al.(2018)	Retrospective cohort study Cox regression survival analysis	Osteomyelitis (clinical diagnosis)	Surgical debridement, except 9 participants who did not receive surgery
Grossi et al.(2016)	Retrospective cohort study Cox regression survival analysis (univariable)	PJI caused by Gram-negative bacteria	Surgical management with curative intent
*Hoell et al.(2016)*	*Retrospective cohort study*	*PJI of total knee arthroplasty defined by: sinus, >*=*2 positive cultures*	*Two-stage revision arthroplasty*
Jhan et al.(2017)	Retrospective cohort study Cox regression survival analysis	MSIS definition of PJI (Parvizi, 2014)	Two-stage revision arthroplasty

**Table 1:** Continued.

Study	Design	Diagnoses	Treatment
Kandel et al.(2019)	Retrospective multi-centre cohort study Cox regression survival analysis	PJI of total hip or knee arthroplasty defined by MSIS definition (Parvizi, 2014)	Single-stage or two-stage revision arthroplasty
Kheir et al.(2018)	Retrospective multi-centre cohort study Logistic regression	MSIS definition of PJI (Parvizi, 2014)	Surgical management
Kurd et al.(2010)	Prospective arthroplasty database cohort study	PJI defined by any of: positive pre-operative or intra-operative microbiology, abscess or sinus	Two-stage revision arthroplasty
*Lam et al.(2019)*	*Retrospective cohort study*	*Clinical diagnosis of osteomyelitis of the ankle, tibia or fibula, with available outcome data and radiography*	*Surgical debridement with free tissue transfer if required*
Lange et al.(2016)	National patient registry retrospective cohort study; Fine and Gray competing-risk regression survival analysis	Chronic PJI defined by clinical code ICD-10 T84.5, verified manually; treatment code present and at least 4 weeks symptoms	Revision arthroplasty withreimplantation
Ma et al.(2018)	Retrospective cohort study Cox regression survival analysis	PJI of total knee arthroplasty defined by MSIS definition (Parvizi, 2014)	Two-stage revision arthroplasty
*Merlet et al.(2014)*	*Retrospective cohort study*	*Calcaneal osteomyelitis defined by any of: visible bone, radiological abnormality, positive microbiology from bone biopsy*	*Not described*
Mortazavi et al. (2011)	Prospective arthroplasty database cohort study Logistic regression	PJI; definition not described	Two-stage revision arthroplasty
Petis et al.(2019)	Retrospective arthroplasty databasecohort study Cox proportional hazard survivalanalysis	PJI of primary arthroplasty, MSIS definition (Parvizi, 2014)	Two-stage revision arthroplasty
*Russell et al.(2020)*	*Retrospective cohort*	*Osteomyelitis of pelvic bones complicating pressure ulcers, defined by clinical diagnosis with radiographic changes*	*First debridement surgery*
Sabry et al.(2014)	Retrospective cohort Cox proportional hazard survivalanalysis	PJI of total knee arthroplasty defined by any of: sinus, purulence, positive microbiology, synovial leukocytosis, positive histopathology	Two-stage revision arthroplasty
Sakellariou et al. (2015)	Retrospective cohort study Logistic regression	PJI of primary knee arthroplasty defined by clinical diagnosis	Two-stage revision arthroplasty
Son et al.(2017)	Medicare Inpatient Claims Database retrospective cohort study Cox proportional hazard survivalanalysis	Participants > 65 years old PJI of total knee arthroplasty defined by ICD-9 code and CPT procedure codes	Not described
Souza Jorgeet al. (2017)	Retrospective cohort study Logistic regression	Fracture-related infection defined using CDC NHSN criteria (CDC, 2020) in participants aged >= 12 years	First surgical debridement
Q. Wang et al. (2019)	Retrospective cohort study Cox proportional hazard survivalanalysis	MSIS definition of PJI (Parvizi, 2014)	Two-stage revision arthroplasty

**Table 1:** Continued.

Study	Design	Diagnoses	Treatment
S. H. Wang et al. (2019)	Retrospective cohort study Logistic regression	MSIS definition of PJI (Parvizi, 2014)	Two-stage revision arthroplasty
Watts et al.(2014)	Retrospective cohort study Cox proportional hazards survivalanalysis	Prosthetic joint infection of total kneearthroplasty defined by any of: purulence, sinus, positive microbiology orhistology	Two-stage revision arthroplasty
			

				Number of
				participants
	Minimum		Number of	experiencing
Study	follow-up period	Outcome definition	participants	outcome (%)
*Ahmad et al.(2019)*	>= *24 months*	*Patients who did not have “successful re-implantation of a revision hip arthroplasty i.e. without functional failure”*	*67, of whom 2 lost to follow-up*	*16 (24 %)*
*Anderson etal. (2018)*	*Not described*	*Consensus definition of PJI treatment failure (Diaz-Ledezma, 2013)*	*60*	*48 (80 %)*
Barshes et al.(2016)	Minimum 4 d; median 8 months	Unanticipated resection of additional bone in contiguous area, or major (above ankle) amputation	184	53 (29 %)
Barton et al.(2019)	>= 24 months; mean 56 months	Failure of infection-free reimplantation arthroplasty; failure to undergo reimplantation arthroplasty; diagnosis of PJI (MSIS criteria, Parvizi, 2014) or further revision surgery following reimplantation arthroplasty	89	37 (42 %)
Bejon et al. (2010)	Mean 69 months	Failure to undergo reimplantation arthroplasty; sinus recurrence, amputation and further surgical treatment following reimplantation arthroplasty	152	26 (17 %)
Cancienne etal. (2017)	>= 12 months	In hospital mortality; repeat debridement without reimplantation; amputation; arthrodesis; retained spacer	7146	2845 (40 %)
Cancienne etal. (2018)	>= 12 months	In hospital mortality; repeat debridement without reimplantation; amputation; arthrodesis; retained spacer	18533	7113 (38 %)
*Carrega et al. (2020)*	>= *24 months; median 44 months*	*Treatment failure (not defined)*	*93*	*14 (15 %)*
Cha et al.(2015)	>= 24 months; mean 30 months	Treatment failure (not defined)	76	18 (24 %)
Chen et al.(2017)	>= 36 months; mean 116 months	Antimicrobial suppression or further surgical treatment	155	13 (8 %)
Cochran et al.(2016)	12 months primary endpoint	Procedure codes indicating surgical treatment for PJI	16622	4322 (26 %)
*Cook et al.(2007)*	>= *12 months; mean 32 months*	*Treatment failure defined by calcaneal wound not fully epithelialised at follow-up*	*50*	*8 (16 %)*
*Faschingbauer et al. (2019)*	>= *24 months*	*Treatment failure defined by MSIS criteria for PJI diagnosis (Parvizi, 2014)*	*96*	*18 (19 %)*
*Ford et al.(2018)*	*Mean 40 months*	*Consensus definition of PJI treatment failure (Diaz-Ledezma, 2013)*	*80*	*14 (18 %)*

**Table 1:** Continued.

				Number of
				participants
	Minimum		Number of	experiencing
Study	follow-up period	Outcome definition	participants	outcome (%)
Garcia delPozo et al. (2018)	>= 12 months; mean 67 months	Treatment failure (not defined)	116	26 (24 %)
Grossi et al.(2016)	>= 24 months	Requirement for further surgery, further antimicrobial therapy, or mortality	76	16 (21 %)
*Hoell et al.(2016)*	>= *16 months; mean 49 months*	*Further surgical intervention*	*59*	*18 (31 %)*
Jhan et al.(2017)	>= 24 months; mean 68 months	Treatment failure, including further surgery or antimicrobial therapy	62	11 (18 %)
Kandel et al.(2019)	24 months primary endpoint	Treatment failure, including excision arthroplasty, amputation, mortality within 30 d or further antimicrobial therapy	533	132 (25 %)
Kheir et al.(2018)	>= 12 months	Consensus definition of PJI treatment failure (Diaz-Ledezma, 2013)	1438	543 (38 %)
Kurd et al.(2010)	>= 24 months; mean 35 months	Treatment failure (not defined)	96	26 (27 %)
*Lam et al.(2019)*	>= *12 months; mean 47 months*	*Clinical diagnosis of infection recurrence*	*67*	*6 (9 %)*
Lange et al.(2016)	Not described	Any of: sinus; positive microbiology in >= 3 surgical specimens or joint fluid; visible purulence; radiological changes; abnormal CRP or ESR	117	17 (15 %)
Ma et al.(2018)	>= 24 months	Consensus definition of PJI treatment failure (Diaz-Ledezma, 2013)	108	16 (15 %)
*Merlet et al.(2014)*	>= *12 months*	*Treatment failure defined by calcaneal wound not fully epithelialised at follow-up, or clinical concern*	*42*	*14 (33 %)*
Mortazavi etal. (2011)	>= 24 months; mean 46 months	Treatment failure defined by any of: positive microbiology, purulence, sinus, abnormal CRP or ESR	137	33 (24 %)
Petis et al.(2019)	>= 24 months; mean 168 months	Treatment failure defined by re-operation or antimicrobial suppression for >= 6 months	245	41 (17 %)
*Russell et al.(2020)*	*Mean 44 months*	*Treatment failure defined by further surgery, re-admission for intravenous antimicrobial therapy, or positive bone microbiology*	*35*	*24 (69 %)*
Sabry et al.(2014)	>= 2 months; mean 40 months	Treatment failure defined by further surgery for microbiologically confirmed recurrence	314	105 (33 %)
Sakellariou et al. (2015)	>= 24 months	Treatment failure defined by any one of: abnormal ESR or CRP; positive microbiology; purulence or sinus	118	15 (13 %)
Son et al.(2017)	Not described	ICD-9 procedure codes for above-knee amputation or arthrodesis	44 466	14 625 (30 %)
Souza Jorgeet al. (2017)	Not described	Treatment failure defined by any of: clinical, laboratory or radiological signs of infection, surgical or antimicrobial therapy after completion of index treatment	193	38 (20 %)

**Table 1:** Continued.

				Number of
				participants
	Minimum		Number of	experiencing
Study	follow-up period	Outcome definition	participants	outcome (%)
Q. Wang et al. (2019)	Mean 66 months	Consensus definition of PJI treatment failure (Diaz-Ledezma, 2013)	341	98 (29 %)
S. H. Wang et al. (2019)	>= 12 months	Consensus definition of PJI treatment failure (Diaz-Ledezma, 2013)	616	132 (21 %)
Watts et al.(2014)	Not described	Revision surgery, clinical diagnosis of reinfection; PROMS reported	111	20 (18 %)

## 3 Results

Figure 1 describes the selection and inclusion of studies in this systematic review.

In total, 35 studies met eligibility criteria and were included, involving 92 693 participants recruited between 1987 and 2018. All studies were retrospective analyses of participants receiving treatment in secondary and tertiary centres. The geographical distribution of reported studies included 18 from the USA, 3 from Taiwan, 2 each from the UK, Germany, France and China, and 1 each from Canada, Brazil, Spain, Italy, South Korea and Denmark.

**Figure 2:**
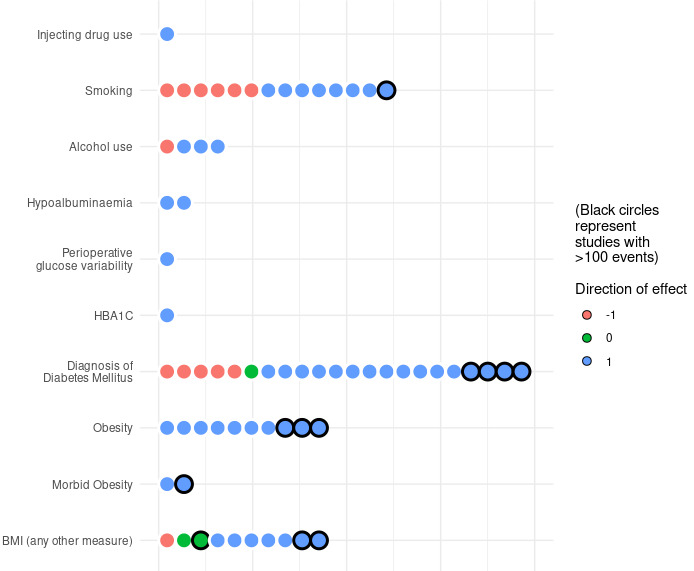
Direction of association for grouped variables representing prognostic factors of interest identified from included studies. Each circle represents the direction of association for one variable in one study. Note that all studies were assessed at a high risk of bias.

Table 1 describes characteristics of included participants and their treatment. No studies performed multivariable modelling using PROMS, and only one study investigated the association between a prognostic factor and PROMS (Watts et al., 2014). Twenty-five studies included a multivariable analysis of treatment outcomes, while nine studies reported univariable analyses only. No studies published a model equation, though this was estimated from Kheir et al. (2018).

Statistical synthesis was not undertaken as all studies were assessed to be at high risk of bias, predominantly due to lack of information on how risk factors were measured and blinding between risk factor and outcome measurement (Hayden et al., 2013). Studies did not include the same prognostic factors of interest in adjusted analyses. Sample size was assessed for each study, and model overfitting resulting from the inclusion of too many prognostic variables contributed to the assessment of risk of bias (Moons et al., 2014).

Two studies fit the definition of risk prediction modelling studies (Sabry et al., 2014; Kheir et al., 2017). Neither study performed internal or external validation, nor was an external validation of these risk prediction models identified during this systematic review. Both studies were assessed against the PROBAST tool to have an overall high risk of bias (Table 2).

**Table 2:** Risk of bias assessment for included risk prediction modelling studies according to the PROBAST tool (Wolff et al., 2019).

Study	Participants	Predictors	Outcome	Analysis	Overall risk ofbias
Kheir et al. (2018)	Retrospective studyof participants in aprospective cohort.Standard diagnosticdefinition. Patients with missingdata excluded; 1438 included, 285 excluded(16.5 %). Some participantsunderwent incision anddrainage, with a lowersuccess rate (47.5 %compared to > 70 %)	Smoking and injecting drug use were retrospectively recordedfrom anaesthetic history. Some patients were telephoned torecord missing data. No blinding described. Organism, choice of surgery and synovial fluid markersmay not be knownpre-operatively	Standard outcomedefinition for treatmentfailure was applied(Norman et al., 2008). No blinding describedbetween predictors and outcome. At least 12 monthsfollow-up for eachparticipant included.	10 predictors chosen basedon the Akaike informationcriterion. Calibration curvesreported. Discriminationassessed based on AUC(0.69, 95 % CI 0.65–0.73). Classification not reported. Shrinkage for overfittingnot reported. Internal and externalvalidation not reported.	High
Sabry et al. (2014)	Retrospective cohortstudy. Non-standarddiagnostic definition. Excluded participantswho were lost tofollow-up (1.7 %) anddid not undergo reimplantation of prosthetic joint (9.6 %).	Predictors and outcomes were retrospectively recorded from the clinical record. No blinding described. Multiple imputation for missing predictors and outcomes. Organism and choiceof surgery may not beknown pre-operatively.	Non-standard outcomedefinition. Antibiotic suppressionfor recurrent infectionnot included in definedtreatment failure. No blinding describedbetween predictors andoutcome. Minimum follow-up59 d.	Not clear how predictorswere selected. Classification notdescribed. Discrimination assessedbased on internal bootstrap resampling AUC (0.773). Competing risks forprimary outcome notdiscussed. External validation notreported.	High

Kheir et al. (2018) presented a logistic regression model for treatment failure as a risk calculator based on points for baseline prognostic factors, following surgery for PJI. They included body mass index (BMI) and ever smoking in the prognostic calculator, as well as a number of non-modifiable factors. The largest estimated odds ratios of treatment failure for dichotomous variables were conferred by treatment with irrigation and debridement rather than implant revision (OR 2.48) and a history of myocardial infarction (OR 1.57). For every unit increase in BMI, the odds of treatment failure were estimated to increase 1.02-fold. For participants who had ever smoked, the odds of treatment failure were estimated to increase 1.2-fold.

Sabry et al. (2014) created a nomogram, based on a logistic regression model, for predicting treatment failure following two-stage revision arthroplasty for knee PJI. The prognostic variables included BMI, but adjusted odds ratios for the variables of interest were not reported.

Of 25 studies that reported multivariable statistical analyses, 20 did not have an adequate sample size to model the number of prognostic factors, according to the proportion of participants with the outcome and assuming $R^{2}=0.25$ (Ensor et al., 2019). In 30 of 35 studies that included univariable prognostic modelling, fewer than 10 outcome events were included per prognostic variable of interest. In 14 studies, the number of prognostic variables described was equal to, or more than, the total number of participants experiencing the treatment outcome of interest.

A summary of the direction of association reported for potentially modifiable prognostic factors and related variables is shown in Fig. 2. One study reported unadjusted and adjusted odds ratios (ORs) for treatment failure in study participants with a history of injecting drug use (Jhan et al., 2017). No studies identified the association of nutritional status with the risk of treatment failure for bone and joint infection. Pre-operative albumin level, a surrogate marker used to assess nutritional status, was included in three studies, of which two studies reported a negative association between measured serum albumin and treatment failure. Two studies reported variables relating to glycaemic control (peri-operative blood glucose variability and HbA1C) (Barshes et al., 2016; S. H. Wang et al., 2019).

A greater proportion of larger studies, with more than 100 participants with the primary outcome, reported higher odds of treatment failure (positive direction of association) for smoking, a diagnosis of diabetes mellitus, and BMI when compared to smaller studies. The ratio of studies reporting a positive direction of association was greatest for glycaemic control and HbA1C (100 %, 2/2), albumin (100 %, 2/2) and BMI-related variables (86 %, 19/22), followed by alcohol intake (75 %, 3/4) and smoking (57 %, 8/14). One study reported a positive direction of association for injecting drug use (100 %, 1/1). This is summarised in Fig. 2.

Many prognostic studies reported the results of a hypothesis test of the direction and magnitude of the association between modifiable prognostic factors and treatment failure following surgery. The ratio of studies reporting a significant (P< 0.05) positive association was greatest for albumin (67 %, 2/3) and variables relating to BMI (42 %, 11/26), followed by alcohol intake variables (25 %, 2/8), diabetes diagnosis (20 %, 6/30), smoking (17 %, 4/24), glycaemic control (0/3) and injecting drug use (0/4).

**Figure 3:**
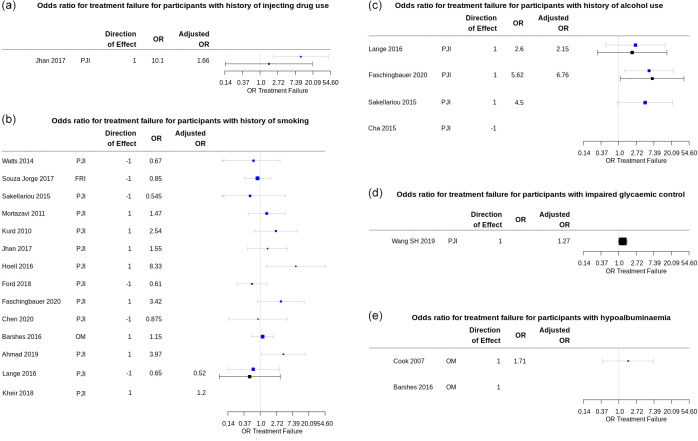
Summary plots of the odds ratio (OR) for treatment failure in participants with a modifiable risk factor of interest reported in included prognostic studies. Univariable ORs are shown in blue; multivariable (adjusted) ORs are shown in black. Plots produced using package forestplot for R (Gordon, 2020). **(a)** Univariable and adjusted ORs for treatment failure for participants with a history of injecting drug use. The adjusted OR from Jhan et al. (2017) is for Cox proportional hazard regression analysis for treatment failure, adjusted for BMI, liver cirrhosis, microbiology investigations, the presence of a sinus tract, repeated surgical debridement, and operating time > 4 h. **(b)** Univariable and adjusted OR for treatment failure for participants with a history of smoking. Adjusted OR from Lange et al. (2016) is for treatment failure at 12 months in all study participants, adjusted for age, sex, American Society for Anaesthesiology (ASA) score, diagnosis of diabetes mellitus, BMI, and alcohol use, using logistic regression modelling from the published data. Confidence intervals for adjusted OR in Kheir et al. (2018), calculated from the risk prediction model-scoring equation, were not reported. **(c)** Univariable and adjusted ORs for treatment failure for participants with a history of alcohol use or dependence. Adjusted OR from Lange et al. (2016) is for treatment failure at 12 months in all study participants, adjusted for age, sex, ASA score, diagnosis of diabetes mellitus, BMI, and smoking, using logistic regression modelling from the published data. Adjusted OR from Faschingbauer et al. (2020) was adjusted for surgical approach (two-stage revision), additional revision between first- and second-stage surgery, and number of prior surgical procedures. **(d)** Adjusted OR for peri-operative blood glucose variability reported in S. H. Wang et al. (2019) was adjusted for “all confounders”, which is understood to include age, sex, BMI, joint involvement, Charlson comorbidity index, diagnosis of diabetes mellitus, rheumatic disease, index surgery (primary or revision), debridement and irrigation before spacer placement, and spacer exchange, as reported in the study methods. **(e)** Univariable OR for treatment failure for participants with hypoalbuminemia (serum albumin 2.2 to 3.0 g L${}^{-1}$) reported in Cook et al. (2007).

Figure 3 shows unadjusted and adjusted odds ratios for treatment failure after surgery for participants with the modifiable prognostic factors of interest.

The identification and selection of participants were reported in 21 of 35 studies. Study setting and recruitment dates were reported in all studies. Study sample size was often inadequate relative to the number of prognostic factors investigated. This contributed to prognostic model overfitting, and thus most studies were considered at a high risk of bias. Participant exclusion following enrolment was often reported, contributing to bias in some studies. For example, a participant who died of severe infection that may have been related to treatment failure was excluded from the analysis (Cha, 2015). Study follow-up duration was well described, but loss to follow-up was up to 17 % and may have been higher in participants who had not experienced treatment failure (Kheir et al., 2017).

No studies described the measurement of prognostic factors in sufficient detail to allow replication. The source, definition, independence, and particularly timing of the prognostic factor were not specified. This could have been remedied if study protocols had been published. Additionally, few studies described the handling of missing values; only one study reported using multiple imputation (Sabry et al., 2014).

## 4 Discussion

This systematic review identified prognostic studies aiming to predict the outcome of surgery for bone and joint infection that included modifiable prognostic factors. Few studies measured nutrition, peri-operative glycaemic control, or substance use for prognosis.

The two clinical prediction modelling studies had a high risk of bias according to independent review by two investigators using the PROBAST tool. Furthermore, the overall risk of bias of the other prognostic studies identified, using the QUIPS tool, was also moderate or high. The studies suggest that modifiable factors, including smoking, glycaemic control, and alcohol intake, predict higher odds of treatment failure. The prognostic value from these factors appeared to be outweighed by others, including diagnosed cardiac, hepatic or renal failure, the number and history of prior revision surgery for prosthetic joint infection, the surgical approach, and soft tissue coverage. No clinical prediction studies were identified that included participants with osteomyelitis or fracture-related infection (FRI).

Modelling prognosis in orthopaedic infection is complicated. Firstly, the treatment pathway may be difficult to standardise. This is illustrated by Anderson et al. (2018), who predicted successful treatment for patients with total knee arthroplasty infection that required extensor mechanism reconstruction, and by Barton et al. (2019), describing attrition in participants intending to receive two-stage revision hip or knee arthroplasty. In the first study, six participants did not start the intended treatment, and those who did had between 1 and 14 surgical procedures. In the second study, only 68 % of patients completed the intended treatment. The modes of treatment failure were functionally different and hence described as separate outcomes in the study report. Complex treatment pathways make it particularly challenging to select an a priori outcome for patients treated for orthopaedic infection. Different definitions of recurrence made it difficult to compare studies.

Treatment decisions are not independent of prognostic factors for treatment failure. Competing risks must be accounted for in prognostic studies. Mortality, amputation and long-term antimicrobial suppression, which were often not defined as treatment failure, are competing risks. Several studies reported a greater number of participant deaths than treatment failures (Chen et al., 2015; Russell and Tsang, 2020). In one study, participants did not undergo reimplantation surgery based on a prognostic factor and so were excluded from analysis (Q. Wang et al., 2019).

Without an explicit definition of how and when a prognostic factor was recorded, its use in risk prediction will not allow an accurate estimate of prognosis. “Smoking” may refer to a diagnostic code in the secondary care record, a note on an anaesthetic chart (Kheir et al., 2017), directly asking a patient, or confirming with carbon monoxide measurement. It may refer to smoking at any time in the past (Kheir et al., 2017), a particular minimum pack-year history (Barshes et al., 2016), pre-operative smoking (Barton et al., 2019), or ongoing smoking after surgery. The timing and dose are important – quitting 4 weeks before surgery appears to be associated with improved healing and a lower risk of primary osteomyelitis (Truntzer et al., 2014). Only one study reported the timing of a prognostic factor recording at least 12 months before the outcome (Son et al., 2017). The heterogeneity in OR of treatment failure for participants with a history of smoking may reflect these differences.

Limitations of the review process include language restriction due to the databases included (though studies published in German and French were reviewed), limitations of the search strategy, and amalgamation of orthopaedic infections affecting differing patient populations and carrying differing prognoses. Additionally, it is possible that some risk prediction modelling studies have been classified as prognostic studies. It can be challenging to infer from published reports whether a study is aiming at prognostic modelling or epidemiology, due to the lack of clarity in the term “risk factor” and the similar statistical methods used in both types of study. It is important that studies state the underlying assumptions of their modelling approach and describe whether the aim is prediction or causal inference (Schooling and Jones, 2018).

The modifiable prognostic factors considered in this systematic review are complex variables that may not have a linear relationship with prognosis and may not be independent. The association of social determinants of health with prognosis, access to healthcare, and the choice of treatments may be relevant to the external validity of prognostic models in different patient groups. Some studies reported health insurance as a surrogate marker for socio-economic status, but this was not included in reported clinical prediction models (Son et al., 2017; Barton et al., 2019; Q. Wang et al., 2019). Barshes et al. (2016) was the only study to assess participants' housing status and found a positive association between homelessness and treatment failure (Barshes et al., 2016).

Finally, independent external validation of prognostic models benefits from the publication of model equations to enable assessment of their calibration and discrimination in new populations. Neither of the risk prediction modelling studies included in this review published the prognostic model equation, so it was estimated from available data (Sabry et al., 2014; Kheir et al., 2017). Improvement in the quality of prognostic study reporting in orthopaedic infection will aid shared decision-making with patients prior to major surgery.

## Supplement

10.5194/jbji-6-257-2021-supplement The supplement related to this article is available online at: https://doi.org/10.5194/jbji-6-257-2021-supplement.

## Data Availability

Data were manually extracted from published data in studies referenced in this review. R packages (open access) used in the presentation of the extracted data are referenced. Statistical techniques used to synthesise data are described in study methods and more specifically in figure legends to facilitate reproduction of these results.
