# Supplementary material for: Systematic review of risk prediction studies in bone and joint infection: are modifiable prognostic factors useful in predicting recurrence?
Source: J Bone Jt Infect. 2021 Jul 8;6(7):257–71. doi: 10.5194/jbji-6-257-2021 (PMC8283517; doi:10.5194/jbji-6-257-2021)
Supplement: The supplement related to this article is available online at: https://doi.org/10.5194/jbji-6-257-2021-supplement. [file jbji-6-257-supplement.pdf]

Supplement of J. Bone Joint Infect., 6, 257–271, 2021
https://doi.org/10.5194/jbji-6-257-2021-supplement
© Author(s) 2021. CC BY 4.0 License.

Supplement of

Systematic review of risk prediction studies in bone and joint infection: are modifiable prognostic factors useful in predicting recurrence?

Maria Dudareva et al.

Correspondence to: Maria Dudareva (maria.dudareva@ouh.nhs.uk)

The copyright of individual parts of the supplement might differ from the article licence.

Supplementary data
(S1) Search strategy used to identify studies for this systematic review.

(osteomyelitis OR osteitis OR “fracture-related infection” OR “fracture-related infection” OR FRI OR PJI OR “prosthetic joint infection” OR “septic arthroplasty” OR “infected arthroplasty” OR “arthroplasty infection” OR “septic THA” OR “septic TKA” OR “diabetic foot” OR spondylodiscitis OR discitis)
AND
(recurrence OR recurrent OR reinfection OR failure OR failed OR success*)
AND
(surg* OR debride* OR excis* OR revision OR revised OR “single-stage” OR “single stage” OR “first stage” OR “first-stage” OR “two stage” OR “two-stage”)
AND
(risk OR diabet* OR smoking OR smoker* OR alcohol OR nutrition OR malnutrition OR malnourished OR diet* OR glycaemic OR glucose OR HbA1C)

Table S2: Risk of Bias assessment
QUIPS Risk Of Bias assessment of 33 prognostic studies that did not meet the definition of clinical prediction modelling studies.[22] Additional indicator questions were used to address the structure of the data used in prognostic studies and the appropriateness of the study sample size. [21,23,27]

Study	Risk of Bias assessment domains for other prognostic studies							
	Data structure and sample size	Study popu-lation	Study attrition	Prognostic factor measure-ment	Outcome measure-ment	Study confound-ing	Statistical analysis and reporting	Overall risk of bias
Ahmad et al. 2019 [30]	High	High	Mod	High	High	Mod	High	High
Anderson et al. 2018 [31]	High	High	Mod	High	High	High	High	High
Barshes et al. 2016 [32]	High	High	Mod	High	High	Low	High	High
Barton et al. 2019 [33]	High	Mod	High	High	High	Mod	High	High
Bejon et al. 2010 [34]	High	High	Mod	Mod	High	Mod	High	High
Cancienne et al. 2017 [35]	High	High	High	High	High	High	High	High
Cancienne et al. 2018 [36]	High	High	High	High	High	High	High	High
Carrega et al. 2020 [37]	High	High	High	High	High	High	High	High
Cha et al.	High	High	High	High	High	High	High	High

2015 [38]								
Chen et al. 2017 [39]	High	Mod	High	High	High	High	High	High
Cochran et al. 2016 [40]	High	Mod	High	High	High	High	High	High
Cook et al. 2007 [41]	High	High	High	High	High	High	High	High
Faschingbauer et al. 2019 [42]	High	High	High	High	High	Mod	High	High
Ford et al. 2018 [43]	High	High	High	High	High	High	High	High
Garcia del Pozo et al. 2018 [44]	High	High	High	High	High	High	High	High
Grossi et al. 2016 [45]	High	High	High	High	High	Mod	High	High
Hoell et al. 2016 [46]	High	High	Low	High	High	Mod	High	High
Jhan et al. 2017 [47]	High	High	High	High	High	Mod	High	High
Kandel et al. 2019 [48]	High	Mod	High	High	High	Mod	High	High
Kurd et al. 2010 [50]	High	High	High	High	High	Mod	High	High
Lam et al. 2019 [51]	High	Mod	Low	High	High	High	High	High
Lange et al. 2016 [52]	Mod	High	Mod	High	High	Mod	High	High
Ma et al. 2018 [53]	High	Low	High	High	High	Mod	High	High
Merlet et al. 2014 [54]	High	Low	High	High	High	Mod	High	High
Mortazavi et al. 2011 [55]	Mod	Low	High	High	High	Mod	High	High
Petis et al. 2019 [56]	High	Mod	High	High	High	Mod	High	High
Russell & Tsang et al. 2020 [57]	High	Mod	High	High	High	High	High	High
Sakellariou et al. 2015 [59]	High	High	High	High	High	Mod	Mod	High
Son et al. 2017 [60]	High	High	High	High	High	Mod	High	High

Souza Jorge et al. 2017 [61]	High	High	Mod	High	High	High	High	High
Wang Q et al. 2019 [62]	High	Low	High	Mod	High	Low	Mod	High
Wang SH et al. 2019 [63]	High	Low	High	High	High	Mod	High	High
Watts et al. 2014 [64]	Mod	Low	High	High	High	Mod	High	High
